# HIV-1 Genetic Diversity and Transmitted Drug Resistance Mutations in ART-Naïve Individuals in South Korea from 2021 to 2024

**DOI:** 10.3390/v17060832

**Published:** 2025-06-09

**Authors:** Gayeong Kim, Eun Ji Kim, Min-Seong Kim, Seolhui Kim, Heui Man Kim, Myung-Guk Han, Jin-Sook Wang

**Affiliations:** Division of Viral Diseases, Department of Laboratory Diagnosis and Analysis, Korea Disease Control and Prevention Agency, Cheongju-si 28159, Republic of Korea; gykim3503@korea.kr (G.K.);

**Keywords:** drug resistance, transmitted drug resistance mutations, human immunodeficiency virus, HIV genetic diversity, antiretroviral therapy

## Abstract

In this study, we investigated the proportion of transmitted drug resistance (TDR) mutations and human immunodeficiency virus (HIV)-1 subtypes among 487 antiretroviral therapy (ART)-naïve individuals in South Korea from 2021 to 2024 to inform more effective treatment strategies. Consistent with previous reports, subtype B was most prevalent among HIV-1 subtypes at 50.7%; however, its proportion decreased annually (*p* = 0.047). Various subtypes of circulating recombinant forms (CRFs) were analyzed in this study, resulting in high genetic diversity. The subtype distributions of Korean and non-Korean patients differed, with subtype B (53.7%) and CRF01_AE (34.4%) being dominant in the former and latter, respectively. TDR across antiretroviral drug classes was approximately 3.5% in South Korea. Non-nucleoside reverse transcriptase inhibitors elicited the greatest drug resistance, which increased from 2021 to 2023, with a slight decrease in 2024. The integrase strand transfer inhibitor drugs, elvitegravir and raltegravir, most frequently exhibited high resistance scores. We provide a comprehensive overview of the HIV-1 genetic distribution and TDR patterns in South Korea from 2021 to 2024. Within the broader context of HIV-1 epidemiology in Asia and the Pacific, the findings contribute to a comprehensive understanding of the global distribution of HIV-1 resistance and genotypes, enabling the development of effective interventions.

## 1. Introduction

In 2023, 39.9 million people worldwide (6.7 million in Asia and the Pacific) were living with the human immunodeficiency virus (HIV) [1]. According to the Joint United Nations Programme on HIV/AIDS, 30.7 million people with HIV (PWH) had been treated with antiretroviral (ARV) drugs [1]. The cumulative number of PWH in South Korea was 19,745 as of 2023 [2].

PWH need to undergo lifelong antiretroviral therapy (ART) to control their viral load [3]. However, ART can induce drug resistance mutations (DRMs), which cause viral rebound and treatment failure. Drug resistance can be transmitted to treatment-naïve individuals, and these transferred drug resistance mutations (TDRMs) spread throughout the population over time [4]. Therefore, understanding the prevalence patterns of TDRMs, as well as the HIV genetic diversity, may inform the selection of ARV drugs [5]. Studies performed on newly diagnosed PWH in South Korea have elucidated the pattern of TDRMs and the HIV-1 genetic diversity within the population. Several studies have highlighted the importance of analyzing the TDRMs within HIV-1 subtypes. A survey of PWH in Italy from 2004 to 2012 demonstrated the interplay between HIV genetic diversity and TDRMs [6].

In South Korea, subtype B was the dominant subtype, accounting for 70.7% of the analyzed samples between 2018 and 2019 [7]. However, this prevalence sharply decreased to 42.1% in 2023, whereas that of recombinant forms increased to 53.4% [8]. Previous studies have shown that although HIV-1 subtype B remains predominant among PWH in Korea, the proportion of non-B subtypes has been increasing in recent years, affecting the overall subtype distribution. Non-B subtypes are not limited to infections acquired overseas but are increasingly spreading across various transmission groups, including men who have sex with men (MSM) [9]. Furthermore, genotypic analysis conducted among specific occupational groups showed a higher prevalence of non-B subtypes in individuals with frequent international exposure [10]. This shift may be attributed to increased international mobility and the introduction of diverse population groups, contributing to the rise in non-B subtypes and circulating recombinant forms (CRFs).

Extensive genetic diversity is a major challenge to HIV vaccine development. Although HIV-1 is the most common type of this virus, global diversity has increased owing to changes in country-level distributions of HIV-1 variants [11]. The global genetic diversity of HIV-1 is steadily increasing, with distinct subtype distributions observed across different countries. Given the genetic differences among HIV-1 subtypes and their varying patterns of global dissemination, it is conceivable that subtype-specific HIV-1 vaccines may be required for individual countries [11,12]. Moreover, the genetic variability of the virus influences both the clinical progression of infection and the effectiveness of antiretroviral therapy. Specifically, certain subtypes are associated with distinct mutation patterns, which can impact drug susceptibility. Therefore, understanding and monitoring the distribution of HIV-1 genotypes is essential for informing public health strategies and optimizing treatment approaches [13,14].

In light of these observations, we analyzed the TDRMs and the genetic distribution among newly diagnosed people with HIV (PWH) over the last 4 years. This study was aimed at providing insights into the current status of the HIV-1 epidemic in South Korea based on the TDRM patterns, HIV-1 genetic diversity, and differences in subtypes of Korean and non-Korean patients.

## 2. Materials and Methods

### 2.1. Study Population and Design

In South Korea, HIV confirmatory testing is conducted by the Korea Disease Control and Prevention Agency (KDCA) and 17 provincial Institutes of Health and Environment. All positive specimens nationwide are sent to the KDCA for surveillance to monitor recent HIV infection [15]. Demographic data were obtained from the Division of HIV/AIDS Prevention and Control at the KDCA. Recently diagnosed PWH who had not been exposed to ART were included. The initial diagnosis for all included PWH was made between January 2021 and October 2024.

### 2.2. RNA Extraction, Polymerase Chain Reaction (PCR) Amplification, Sequencing, and Genome Assembly

Viral RNA was extracted from 140 µL of plasma or serum using the QIAcube extraction system (QIAGEN, Hilden, Germany), according to the manufacturer’s instructions. Reverse transcription and nested PCR were performed to amplify the envelope (*env*), gag protein (*gag*), and pol protein (*pol*) for genotyping and the protease, reverse transcriptase, and integrase genes for drug resistance analysis. Genes were reverse-transcribed and amplified using the PrimeScript™ One Step RT-PCR kit (Takara Bio Inc., Kusatsu, Shiga, Japan), and nested PCR was conducted using the AccuPower ProFi Taq PCR PreMix (Bioneer, Yongin, Gyeonggi-do, Republic of Korea). Sanger sequencing was performed using in-house sequencing primers (Table 1). Sequence quality and the generated consensus sequence alignments were assessed using Geneious Prime software version 2025 (Biomatters Ltd., Auckland, New Zealand).

### 2.3. HIV Sequencing and Subtyping

Sequence fragments were edited and assembled using SeqMan Pro 7.1.0 (44.1). HIV subtypes were analyzed based on sequence identity using the automated HIV DB BLAST tools provided by Los Alamos National Laboratory, USA (https://hiv.lanl.gov/content/sequence/BASIC_BLAST/basic_blast.html; accessed on 8 June 2025) [16]. CRFs were classified according to the nomenclature provided by the Los Alamos HIV Sequence Database. For the purpose of analyzing recombination patterns, CRFs were categorized into three groups: CRF_BC (CRF06_BC, CRF07_BC), CRF_01B (CRF33_01B, CRF51_01B), and CRF_0107 (CRF79_0107, CRF109_0107, CRF123_0107). The analysis was conducted by integrating partial sequences of the *env*, *gag*, and *pol* regions. Subtype assignments were determined based on sequence similarity percentages and the frequency distribution across these regions.

### 2.4. HIV Drug Resistance Analysis

Drug resistance regions, specifically protease, reverse transcriptase, or integrase, were targeted for analysis using the Stanford drug resistance database, following the criteria outlined in the analysis guide provided by the World Health Organization (WHO) [17]. Stanford HIVdb drug resistance algorithm version 9.7 was utilized to evaluate the impact of TDRMs on therapeutic responses. This algorithm assigns a specific score to each detected TDRM and categorizes them into one of five susceptibility levels: a total score of less than 10 is considered susceptible (S), 10 to 14 indicates potential low-level resistance (PLR), 15 to 29 indicates low-level resistance (LR), 30 to 59 indicates intermediate resistance (IR), and over 60 indicates high-level resistance (HR). All currently available drugs were included for analysis. Protease inhibitors (PIs): atazanavir (ATV/r), darunavir (DRV/r), fosamprenavir (FPV/r), indinavir (IDV/r), lopinavir (LPV/r), nelfinavir (NFV), saquinavir (SQV/r), and tipranavir (TPV/r); nucleoside reverse transcriptase inhibitors (NRTIs): abacavir (ABC), zidovudine (AZT), stavudine (D4T), didanosine (DDI), emtricitabine (FTC), lamivudine (3TC), and tenofovir (TDF); non-nucleoside reverse transcriptase inhibitors (NNRTIs): doravirine (DOR), efavirenz (EFV), etravirine (ETR), nevirapine (NVP), and rilpivirine (RPV); integrase strand transfer inhibitors (INSTIs): bictegravir (BIC), cabotegravir (CAB), dolutegravir (DTG), elvitegravir (EVG), and raltegravir (RAL). PLR and accessory mutations for PI and INSTI were excluded from the analysis [18]. A two-tailed *t*-test was performed to evaluate whether drug resistance differed significantly between subtype B and non-B populations. Logistic regression analysis was used to assess the patterns in the prevalence of specific genotypes and drug resistance. The resulting *p*-value was used to determine statistical significance.

### 2.5. Ethical Approval

All the authors hereby declare that all the experiments were reviewed and granted an exemption from ethical approval by the appropriate ethics committee (the KDCA Institutional Review Board Ethics Committee; approval number: KDCA-2025-01-03-PE-01). The requirement for informed consent from study participants was also waived by the KDCA Institutional Review Board.

## 3. Results

### 3.1. Study Population

The study included 487 PWH: 99 from 2021, 143 from 2022, 148 from 2023, and 97 from 2024. According to sex, the study population during this period consisted of an average of 95.5% males, 4.3% females, and 0.2% unknown. Koreans and non-Koreans constituted 87.5% and 12.5%, respectively. The countries of origin for non-Koreans were primarily in Asia and the Americas or were unspecified. Table 2 summarizes the demographic information for the PWH.

### 3.2. Genetic Diversity

**Figure 1:**
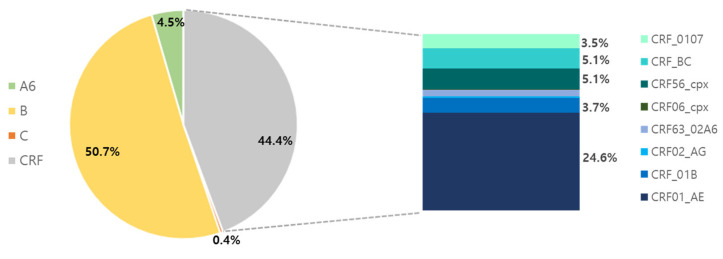
Human immunodeficiency virus (HIV) subtypes were identified in 487 people with HIV (PWH). Proportions <1% are not shown. CRF: Circulating recombinant form.

**Figure 2:**
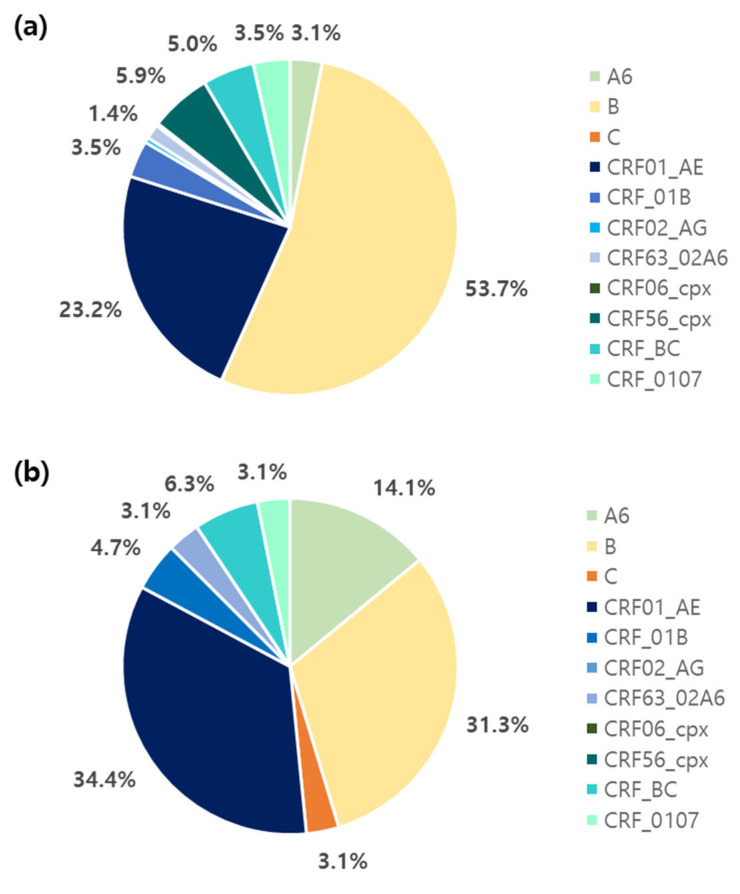
Distribution of HIV subtypes according to ethnic group: (**a**) Koreans and (**b**) non-Koreans. Proportions <1% are not shown. CRF: Circulating recombinant form.

The genotypes of 487 PWH diagnosed from 2021 to 2024 were analyzed. The majority of HIV infections were of subtype B. The proportions of subtypes B, CRFs, A6, and C were 50.7%, 44.4%, 4.5%, and 0.4%, respectively (Figure 1). Among the CRFs, CRF01_AE (24.6%) was the most prevalent, followed by CRF56_cpx (5.1%), CRF_BC (5.1%), CRF_01B (3.7%), CRF_0107 (3.5%), CRF63_02A6 (1.6%), CRF02_AG (0.4%), and CRF06_cpx (0.2%). The subtype patterns were different for Koreans and non-Koreans (Figure 2). Among the 423 Koreans, subtype B (53.7%) was the most prevalent, followed by CRF01_AE (23.2%), CRF56_cpx (5.9%), CRF_BC (5.0%), CRF_01B (3.5%), CRF_0107 (3.5%), A6 (3.1%), CRF63_02A6 (1.4%), CRF02_AG (0.5%), and CRF06_cpx (0.2%). In contrast, CRF01_AE (34.4%) was dominant among non-Koreans, followed by B (31.3%), A6 (14.1%), CRF_BC (6.3%), CRF_01B (4.7%), CRF_0107 (3.1%), CRF63_02A6 (3.1%), and C (3.1%).

### 3.3. Distribution and Characterization of DRMs

**Figure 3:**
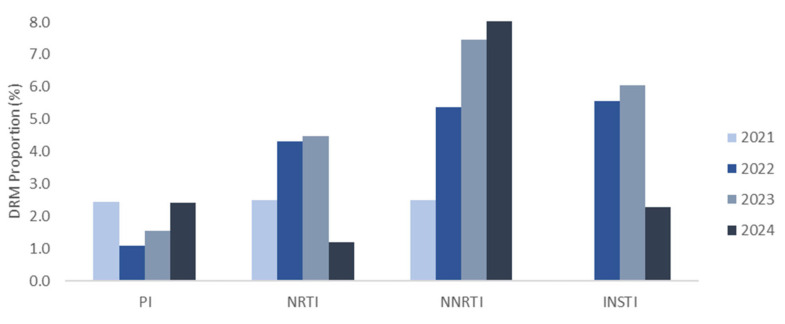
Proportion of drug resistance mutations (DRMs) according to antiretroviral (ARV) drug classes. PI: protease inhibitor; NRTI: nucleoside reverse transcriptase inhibitor; NNRTI: non-nucleoside reverse transcriptase inhibitor; INSTI: integrase strand transfer inhibitor. The HIVdb program, based on the Stanford University HIV Drug Resistance Database, was used to assess DRMs according to ARV class. Accessory mutations were excluded for PI and INSTI.

**Figure 4:**
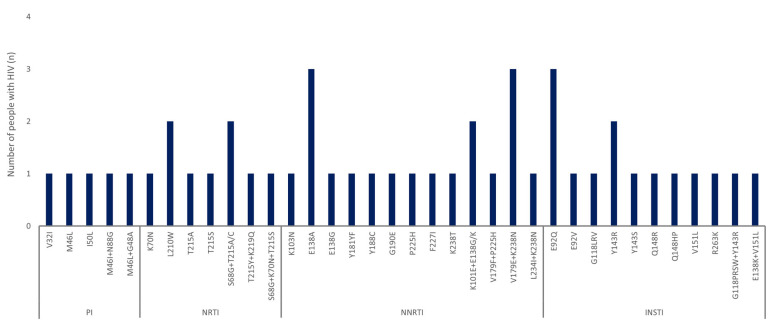
Frequency of mutations associated with resistance to ARV drugs. PI: protease inhibitor; NRTI: nucleoside reverse transcriptase inhibitor; NNRTI: non-nucleoside reverse transcriptase inhibitor; INSTI: integrase strand transfer inhibitor.

**Figure 5:**
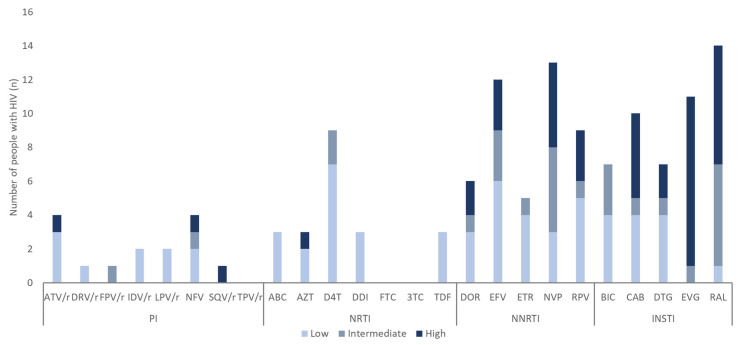
Predicted resistance to antiretroviral drugs for HIV *pol* sequences with DRMs in PWH. PIs: atazanavir (ATV/r), darunavir (DRV/r), fosamprenavir (FPV/r), indinavir (IDV/r), lopinavir (LPV/r), nelfinavir (NFV), saquinavir (SQV/r), and tipranavir (TPV/r); NRTIs: abacavir (ABC), zidovudine (AZT), stavudine (D4T), didanosine (DDI), emtricitabine (FTC), lamivudine (3TC), and tenofovir (TDF); NNRTIs: doravirine (DOR), efavirenz (EFV), etravirine (ETR), nevirapine (NVP), and rilpivirine (RPV); INSTIs: bictegravir (BIC), cabotegravir (CAB), dolutegravir (DTG), elvitegravir (EVG), and raltegravir (RAL).

Drug resistance analysis included 417 PWH from 2021 to 2024. The average drug resistance across classes was 1.9%, 4.1%, 4.9%, and 3.3% in 2021, 2022, 2023, and 2024, respectively (Figure 3). Although a slight increasing pattern was observed in the prevalence from 2021 to 2024, it showed no statistically significant pattern (*p* = 0.495). The most frequent single-class mutations were against NNRTIs (18/284, 6.3%), followed by INSTIs (14/364, 3.8%), NRTIs (9/284, 3.2%), and PIs (5/281, 1.8%). The DRM proportion for NNRTIs, which was the highest, increased from 2.5% in 2021 to 5.4% in 2022, 7.5% in 2023, and 8.3% in 2024 (Figure 3). Drug resistance according to the population subtype (B and non-B) showed no statistically significant differences (*p* = 0.823) (Table 3). E138A and E92Q were the most commonly observed mutations against NNRTIs and INSTIs, respectively (Figure 4). Each drug was independently analyzed. The highest cases of drug resistance were against ATV/r and NFV, D4T, NVP, and RAL among PIs, NRTIs, NNRTIs, and INSTIs, respectively (Figure 5). The analysis of the drug resistance scores for these drugs revealed that drug resistance cases for ATV/r, NFV, and D4T were mostly LR; however, NVP and RAL showed IR or HR drug resistance scores in most cases. NVP elicited five cases of IR and HR and three cases of LR. RAL elicited seven cases of HR, followed by six cases of IR and one case of LR. Detailed information on TDRMs according to drug class is shown in Figure 5.

## 4. Discussion

In this study, we analyzed the HIV-1 genetic distribution and ARV drug resistance among recently diagnosed PWH in South Korea from 2021 to 2024. HIV is a persistent virus that exhibits a high degree of genetic diversity. According to the Los Alamos National Laboratory Database, 158 CRFs have been reported (as of 14 October 2024), and this number is continuously increasing [19]. Subtype B was the most prevalent in South Korea; however, its prevalence has steadily decreased from 93.1% in 1992–2012 to 74.6% in 2018–2019 [7,20,21] and 50.7% in 2021–2024 in this study (*p* = 0.047). Among Koreans, subtype B (53.7%) was the most prevalent, followed by CRF01_AE (23.2%). In contrast, CRF01_AE (34.4%) and B (31.3%) were the most common subtypes among non-Koreans. The global trend of genetic distribution across continents remained relatively unchanged from 2010 to 2021. However, there has been an approximately 8% increase in the proportion of CRFs and unique recombinant forms. CRF01_AE (29%) has been the major subtype among those in Asia and the Pacific [22,23]. Specifically, among the non-Korean individuals in this study, those from Asia and the Pacific have the highest proportion, with the subtype distribution reported as CRF01_AE (29%), B (13%), and C (13%) [22]. The recent changes in the subtype pattern of Koreans appear to have been influenced by the epidemiological trends of neighboring countries. The distribution patterns of subtypes among non-Koreans are primarily associated with the subtypes circulating in their home countries, suggesting that they were likely infected within their home countries. Notably, since 59.4% of the non-Koreans in this study were of Asian nationality, it is important to continuously monitor the molecular genetics of HIV in neighboring countries.

Consistent with previous results [7,8], NNRTIs elicited the highest level of drug resistance, which steadily increased from 2021 to 2024: 2.5% in 2021, 5.4% in 2022, 7.5% in 2023, and 8.3% in 2024 (*p* = 0.025). This study showed that about 3.6% of treatment-naïve PWH were resistant to at least one ARV drug from 2021 to 2024. An analysis of the number of drug resistance cases for a single drug by subtype (B and non-B) showed no significant difference between the groups. According to the clinical treatment guideline for HIV in South Korea, a combination including an INSTI is recommended as the first-line treatment for treatment-naïve patients, followed by a regimen combining an INSTI and NRTI [24]. Currently, there are no cases of drug resistance to both INSTIs and NRTIs for any subtype (B or non-B), suggesting that dual regimens could be viable starting options.

The most prevalent mutation in the NNRTI class was E138A, which conferred LR to RPV. E138A was identified in 2023 and 2024, and all the patients were diagnosed with subtype CRF63_02A6, suggesting that further analysis of this association is needed. Drug resistance to FTC and TDF was also analyzed because RPV has been co-formulated with these drugs for initial therapy [25]. All four cases showed susceptibility to both drugs. The E138A is known to occur in approximately 2–5% of ART-naïve individuals, depending on subtype; however, whether this mutation selectively occurs in NNRTI-treated individuals remains unknown [26]. The most prevalent mutation in the INSTI class was E92Q. The E92Q mutation is recognized as the most common cause of resistance to INSTIs such as EVG and RAL. This mutation has been reported as the most common INSTI mutation at the first time point of treatment failure with INSTI-based ART [27]. Therefore, further research is needed to explore the impact of this mutation on ART.

Although generally excluded from the DRM proportion, PLR should also be considered. In this study, the notable mutations causing PLR were V179D (or E or F) (25 cases) for NNRTIs and E157Q (16 cases) for PIs. V179D (or E) is the most common NNRTI-associated mutation in China and is known to significantly regulate the replication capacity of HIV [28,29,30]. E157Q decreases the integrase enzymatic activity promoted by the R263K substitution, and E157Q–R263K could lead to virological failure upon treatment with DTG [31,32]. These mutations were not detected simultaneously in this study. Therefore, they are not currently at a concerning level, but they must be continuously monitored.

The distribution of resistance scores by drug revealed that HR was most frequently elicited by EVG and RAL, both INSTIs. RAL and EVG were approved by the FDA in 2007 and 2012, respectively, and have been in use for several years [33]. Both these drugs have a low to moderate genetic barrier to resistance; cross-resistance to them has also been observed [34]. Therefore, they are of significant concern.

As a result of increased HIVDR to NNRTIs and subsequent cost implications, the WHO has recommended DTG-based first-line and second-line ART for PWH since 2018 [35,36]. It also recommends high-priority monitoring of HIVDR to DTG by implementing surveys [37]. Additionally, according to the clinical treatment guidelines for HIV in South Korea [24], combinations of antiretroviral drugs such as ABC/3TC/DTG, TAF/FTC/BIC, TAF/FTC+DTG, TAF/FTC+RAL, and 3TC/DTG are recommended. DTG is expected to be commonly prescribed in South Korea. Therefore, the number of drug resistance cases for the DTG during the analyzed period revealed that DTG drug resistance increased from zero cases in 2021 to one and five cases in 2022 and 2023, respectively, but decreased to one case in 2024. The results of this study indicate that, although the frequency of DTG resistance is not yet at a concerning level, continuous monitoring is still necessary.

This study has a few limitations. First, only a limited number of samples were used, including only 10% of the newly diagnosed PWH. Therefore, a more comprehensive data analysis is needed to determine trends across the population. Second, the epidemiological association was limited owing to a lack of certain clinical information, such as the timing of infection, sexual behaviors, risk factors, immune status, history of sexually transmitted and other diseases, and demographic characteristics. Third, a comprehensive analysis of genotypes, including unique recombinant forms (URFs) and drug resistance, is warranted to clarify the mechanisms of genotype diversification and to assess whether resistance arises from antiretroviral exposure. Furthermore, detailed monitoring of the emergence of resistance is necessary to identify the origins and transmission routes of resistance mutations and detect clusters through phylogenetic analysis and related methods [38]. Finally, some foreign individuals were uncertain about whether they were taking any ARV drugs.

Notwithstanding these limitations, this study has several important implications. It provides a comprehensive overview of the distribution of HIV-1 subtypes and patterns in TDR in South Korea from 2021 to 2024. There is no compelling evidence that the HIV subtype needs to be considered when choosing ARV drugs; however, some studies have demonstrated an association between drug resistance and subtypes [39,40,41]. The inclusion criteria ensured that PWH included in this analysis were treatment-naïve at the time of diagnosis, which is crucial for accurately reflecting the proportion and impact of TDRMs in treatment-naïve individuals. The WHO has established HIVResNET to support global drug resistance research on drug resistance [42]. Within the context of HIV epidemiology in Asia and the Pacific, the findings of this study contribute to an in-depth understanding of the global distribution of HIV resistance and genotypes, which should enable the development of more effective response strategies.

## Figures and Tables

**Table 1:** List of primers for sequencing.

	Forward Primers		Reverse Primers	
Gene	Sequences (5′ to 3′)	Position *	Sequences (5′ to 3′)	Position *
*env*	TCTGGGGCATYAARCAGCTC	7933–7952	GGTARMKGAARAGGMACAGGYTCC	8506–8529
*gag*	TACCYATGTTYWCAGCAYTATCAGARGGAGC	1295–1325	TTYCTAGGGGCCCTGCAATTTT	1997–2018
	ATGATGACAGCATGTCAGGGA	1825–1845	TAATGCTTTTATTTTYTCYTCTGTCAATGGC	2621–2651
*pol*	AGACAGGCTAATTTTTTAGGGA	2074–2095	ATGGYTCTTGATAAATTTGATATGTCC	3559–3585
	CAGAGCCAACAGCCCCACCA	2147–2166	CTGCCAGTTCTAGCTCTGCTTC	3441–3462
	TTCRGGATYAGAAGTAAAYATAGTAACAG	4013–4041	TCCCCTAGTGGGATGTGTACT	5202–5222
	TCTACCTGGCATGGGTACCA	4141–4160	CCTAGTGGGATGTGTACTTCTGA	5197–5219

* Nucleotide positions of the 5′ end of each primer in HxB2_K03455.

**Table 2:** Demographics of study population (2021–2024).

Variables		*n* (%) by Collection Year			
		2021	2022	2023	2024
Sex	Male	92 (92.9)	137 (95.8)	142 (95.9)	93 (95.9)
	Female	7 (7.1)	5 (3.5)	6 (4.1)	4 (4.1)
	Unknown	0 (0.0)	1 (0.7)	0 (0.0)	0 (0.0)
Country of origin	Korean	83 (83.8)	121 (84.6)	133 (89.9)	86 (88.7)
	Non-Korean	16 (16.2)	22 (15.4)	15 (10.1)	11 (11.3)

**Table 3:** Proportion of DRMs: single and combination classes differentiated by B and non-B subtypes from 2021 to 2024.

	Antiretroviral Drug Resistance, *n* (%)						
	PI	NRTI	NNRTI	INSTI	PI +NRTI	PI +NNRTI	NRTI +NNRTI
Total	3 (1.1)	5 (1.8)	14 (4.9)	14 (3.8)	1 (0.4)	1 (0.4)	3 (1.1)
B	1 (0.6)	3 (1.8)	7 (4.2)	8 (4.3)	0	0	3 (1.8)
Non-B	2 (1.7)	2 (1.7)	7 (5.9)	6 (3.4)	1 (0.9)	1 (0.9)	0

PI: protease inhibitor; NRTI: nucleoside reverse transcriptase inhibitor; NNRTI: non-nucleoside reverse transcriptase inhibitor; INSTI: integrase strand transfer inhibitor.

## Data Availability

The data used to support this study are available from the corresponding author upon request.

## Abbreviations

The following abbreviations are used in this manuscript:

PWH	People with HIV
ARV	Antiretroviral
ART	Antiretroviral therapy
DRMs	Drug resistance mutations
TDRMs	Transferred drug resistance mutations
KDCA	Korea Disease Control and Prevention Agency
PCR	Polymerase chain reaction
WHO	World Health Organization
S	Susceptible
PLR	Potential low-level resistance
LR	Low-level resistance
IR	Intermediate resistance
HR	High-level resistance
PI	Protease inhibitor
ATV/r	Atazanavir
DRV/r	Darunavir
FPV/r	Fosamprenavir
IDV/r	Indinavir
LPV/r	Lopinavir
NFV	Nelfinavir
SQV/r	Saquinavir
TPV/r	Tipranavir
NRTIs	Nucleoside
ABC	Abacavir
AZT	Zidovudine
D4T	Stavudine
DDI	Didanosine
FTC	Emtricitabine
3TC	Lamivudine
TDF	Tenofovir
NNRTI	Non-nucleoside reverse transcriptase inhibitor
DOR	Doravirine
EFV	Efavirenz
ETR	Etravirine
NVP	Nevirapine
RPV	Rilpivirine
INSTI	Integrase strand transfer inhibitor
BIC	Bictegravir
CAB	Cabotegravir
DTG	Dolutegravir
EVG	Elvitegravir
RAL	Raltegravir
CRF	Circulating recombinant form
